# Poplar *PdPTP1* Gene Negatively Regulates Salt Tolerance by Affecting Ion and ROS Homeostasis in *Populus*

**DOI:** 10.3390/ijms21031065

**Published:** 2020-02-05

**Authors:** Yingying Lu, Wanlong Su, Yu Bao, Shu Wang, Fang He, Dongli Wang, Xiaoqian Yu, Weilun Yin, Chao Liu, Xinli Xia

**Affiliations:** Beijing Advanced Innovation Center for Tree Breeding by Molecular Design, National Engineering Laboratory for Tree Breeding, College of Biological Sciences and Technology, Beijing Forestry University, Beijing 100083, China

**Keywords:** *PdPTP1*, poplar, salinity, ion homeostasis, ROS

## Abstract

High concentrations of Na^+^ in saline soil impair plant growth and agricultural production. Protein tyrosine phosphorylation is crucial in many cellular regulatory mechanisms. However, regulatory mechanisms of plant protein tyrosine phosphatases (PTPs) in controlling responses to abiotic stress remain limited. We report here the identification of a Tyrosine (Tyr)-specific phosphatase, PdPTP1, from NE19 (*Populus nigra* × (*P. deltoides* × *P. nigra*). Transcript levels of *PdPTP1* were upregulated significantly by NaCl treatment and oxidative stress. PdPTP1 was found both in the nucleus and cytoplasm. Under NaCl treatment, transgenic plants overexpressing *PdPTP1* (*OxPdPTP1*) accumulated more Na^+^ and less K^+^. In addition, *OxPdPTP1* poplars accumulated more H_2_O_2_ and O_2_·^−^, which is consistent with the downregulation of enzymatic ROS-scavengers activity. Furthermore, PdPTP1 interacted with PdMAPK3/6 in vivo and in vitro. In conclusion, our findings demonstrate that PdPTP1 functions as a negative regulator of salt tolerance via a mechanism of affecting Na^+^/K^+^ and ROS homeostasis.

## 1. Introduction

Soil salinity is one of the major abiotic stresses that imposes severe threats to the growth and yield of diverse plants. More than 6% of the world’s total land area suffers from overabundance of salt that impedes plant absorption of water and nutrients from the soil [1]. Salt stress is associated with ionic stress, osmotic stress, and secondary oxidative stress in plants [2]. Therefore, primary strategies for plants adapting to salt conditions are to regulate ionic and osmotic homeostasis, as well as detoxification [3]. 

Genetic analyses have indicated that the salt overly sensitive (SOS) pathway is crucial in regulating ionic homeostasis and helps plants adapt to salt stress. In this pathway, exposure to NaCl would elicit a transit increase in cytosolic Ca^2+^ concentrations, which is perceived by a calcium-binding protein, SOS3. SOS3 physically translates to Ser/Thr protein kinase SOS2. The SOS2–SOS3 complex phosphorylate and activate Na^+^/H^+^ exchanger SOS1 located in the plasma membrane, finally transporting Na^+^ out of the cytosol [2,3,4]. In glycophytes, excessive Na^+^ often leads to K^+^ deficiency under salt stress [5]. As a major osmoticum, the maintenance of high cytosolic K^+^ concentrations is crucial for plant salinity tolerance [1,6]. Therefore, maintaining a suitable K^+^/Na^+^ is an important mechanism used by plants to adapt to salt stress. 

Salt-induced secondary oxidative stresses generate ROS, including superoxide anion radical (O_2_·^−^), hydroxyl radicals (·OH), and hydrogen peroxide (H_2_O_2_). Low ROS concentrations have been considered to be a signal that activates specific downstream responses, while high ROS concentrations can have a detrimental effect on DNA, proteins, lipids, and carbohydrates [7]. The harmful effects of ROS generated can be mitigated by numerous enzymatic and nonenzymatic scavengers [2,8]. No-enzymatic antioxidants include ascorbate, glutathione, ascorbic acid, and carotenoids; Enzymatic ROS scavengers include superoxide dismutase (SOD), peroxidase (POD), catalase (CAT), and glutathione reductase (GA) [9].

Mitogen activated protein kinase (MAPK) cascades participate in ionic, osmotic, and oxidative stress signaling in plants [10]. AtMPK6 activated by phosphatidic acid (PA) in response to salt stress phosphorylates and activates the SOS1 Na^+^/H^+^ antiporter activity, which contributes to reduced Na^+^ accumulation [11]. MKK4-MPK3 and MKKK20-MPK6 cascades mediate osmotic stress responses. The *mkk4* and *mkkk20* mutants are hypersensitive to salt stress compared to the wild type (WT) [6]. The Salt Intolerance 1 (SIT1)-MPK3/6 cascade affect ROS and ethylene homeostasis to regulate salt response in rice [12]. MPK3/6 control ROS homeostasis by phosphorylating and activating HEAT SHOCK FACTOR A_4_A (HSFA_4_A) and thereby displayed increased salt resistance [13]. 

Tyr phosphorylation plays a crucial role in animal cells, as it regulates many cellular processes, including division, growth, and differentiation. Tyr phosphorylation in plants, however, was ignored originally due to the lack of a typical tyrosine kinase gene [8]. Even though, several lines of evidence proved the existence of Protein Tyrosine phosphatases (PTPs) in *Arabidopsis* and other species [14,15]. The PTPs superfamily can be divided into two large groups based on phosphoamino acid specificity: phosphotyrosine-specific phosphatases (Tyr-specific PTPs) and dual-specificity phosphatases (DsPTPs) [16]. Notably, at least 38 genes coding Tyr-specific PTPs exist in the human genome, but only one gene is considered to be a member of Tyr-specific PTPs in *Arabidopsis*, *Chlamydomonas reinhardtii*, and *Ostreococcus tauri* [15,17,18], suggesting that plants probably developed a different strategy from mammalians to read the phospho-Tyr signals. 

Studies have shown that PTPs are involved in abiotic stress responses. In *Arabidopsis*, the expression of *AtPTP1*, *a* Tyr specific PTP, was found to be upregulated by salt, and its involvement in oxidative stress signaling was suggested [14,19]. *Arabidopsis* MAPK phosphatase 1 (MKP1), a DsPTP, participates in salt-stress response, which can be indicated by increased resistance of *mkp1* mutant plants to salinity [20]. Furthermore, *mkp1* mutant is also hypersensitive to UV radiation [21]. Moreover, phenylarsine oxide (PAO), a specific inhibitor of PTPs prevents stomatal closure in *Commelina communis* by reopening them [22]. These studies have permitted to have a better view of roles of PTPs in the stress responses. However, these regulatory processes need to be completed in greater detail. 

PTPs are known to be dedicated MAPK phosphatases (MKPs) [23]. Both PTP1 and DsPTPs have been shown experimentally to dephosphorylate MAPKs [24]. PTP1 was found to be able to dephosphorylate and deactivate MPK4 and MPK6 in vitro [25]. Screens for UV-sensitive mutants in *Arabidopsis* identified MKP1 essential for UV resistance can interact with stress-activated MAPK3, MAPK4, and MAPK6 [20]. The constitutive defense response of mutant *mkp1* is strongly enhanced by the *ptp1* null mutation, which could be genetically attributed to increased levels of active MPK3 and MPK6 [26]. Taken together, these data indicate that PTPs may have significant effects on plant stress responses by dephosphorylation of the core kinases in the MAPK cascade. 

In the poplar genome, 38 PTPs superfamily members had been identified by using the complete human genome as reference. Like most of the other plant species, there is also only one Tyr specific phosphatase in poplar [15]. However, the functions of those PTPs in woody plants remain unknown. Here, we isolated and characterized a Tyr-specific PTP gene from NE19, which showed high identity in amino acid sequences to other organisms. Overexpression of *PdPTP1* reduced salinity tolerance in poplar, as a consequence of impairing re-establishment of cellular ionic and ROS homeostasis. Furthermore, we also provided evidence that PdPTP1 interacts with the stress-responsive kinase PdMAPK3/6. This study provides important new information for engineering salt-tolerant poplar.

## 2. Results

### 2.1. PdPTP1 Encodes a Tyr-Specific PTP in Populus

**Figure 1:**
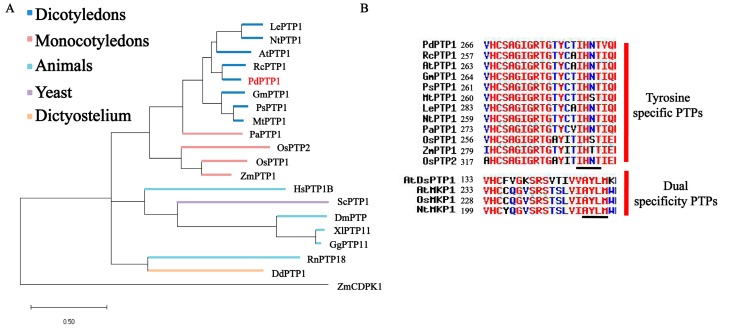
Comparison of PdPTP1 with a number of PTPs amino acid sequence. (**A**) Phylogenetic analysis of PdPTP1 and other PTPs from eukaryotes. Sequences are from Dicotyledons (*A. thaliana* AtPTP1, *Glycine max* GmPTP1, *Ricinus communis* RcPTP1, *Pisum sativum* PsPTP1, *Medicago truncatula* MtPTP1, *Nicotiana tobacum* NtPTP1, *Lycopersicon esculentum* LePTP1), Monocotyledons (*Phalaenopsis amabilis* PaPTP1, *Oryza sativa* OsPTP1, *Oryza sativa* OsPTP2, *Zea mays* ZmPTP1), Yeast (*Saccharomyces cerevisiae* ScPTP1), Animals (*Homo sapiens* HsPTP1B, *Rattus norvegicus* RnPTP18, *Gallus gallus* GgPTP11, *Xenopus laevis* XlPTP11, *Drosophila melanogaster* DmPTP), and Dictyostelium (*Dictyostelium discoideum* DdPTP1). (**B**) Comparisons of the conserved catalytic domains between Tyr-specific and dual-specificity PTPs in plants. The sequence underlined in black designates the putative IHNT domain of Tyr-specific PTPs and MKP domain “AYLM” of dual-specificity PTPs, respectively.

Based on the CDS sequences of the *P. trichocarpa PtPTP1* gene, the *PdPTP1* gene in NE19 was isolated by using the polymerase chain reaction (PCR) approach, using the specific primers (Table S1). The full-length nucleotide sequence of cDNA is 1020 bp that encodes a protein of 339 amino acids. PdPTP1 shares high identity with *Arabidopsis* (*Arabidopsis thaliana*, 65.64%) AtPTP1, soybean (*Glycine max*, 64.51%) GmPTP1, and pea (*Pisum sativum*, 62.82%) PsPTP1, suggesting that PdPTP1 may be a member of Tyr-specific PTPs. The phylogenetic tree of PdPTP1 protein and other PTPs from eukaryotes was constructed (Figure 1A, Table S2). Furthermore, an amino acid sequence alignment of the PdPTP1 with PTPs from plants, human, and rats shows that PdPTP1 contains an amino acid sequence motif “(I/V)HCXAGXXR(S/T)G” characteristic for many active PTPs and the KNRY and the WPD motifs specific to Tyr-specific PTPs (Figure S1). Moreover, PdPTP1 contains the IHNT domain of Tyr-specific PTPs, but not MKP domain “AYLM” of dual-specificity PTPs, by comparing the conserved catalytic domains between Tyr-specific and dual-specificity PTPs in plants (Figure 1B). These results suggested that PdPTP1 is a novel member of the Tyr-specific PTP in poplar.

### 2.2. PdPTP1 Localized to both the Cytosol and Nucleus

**Figure 2:**
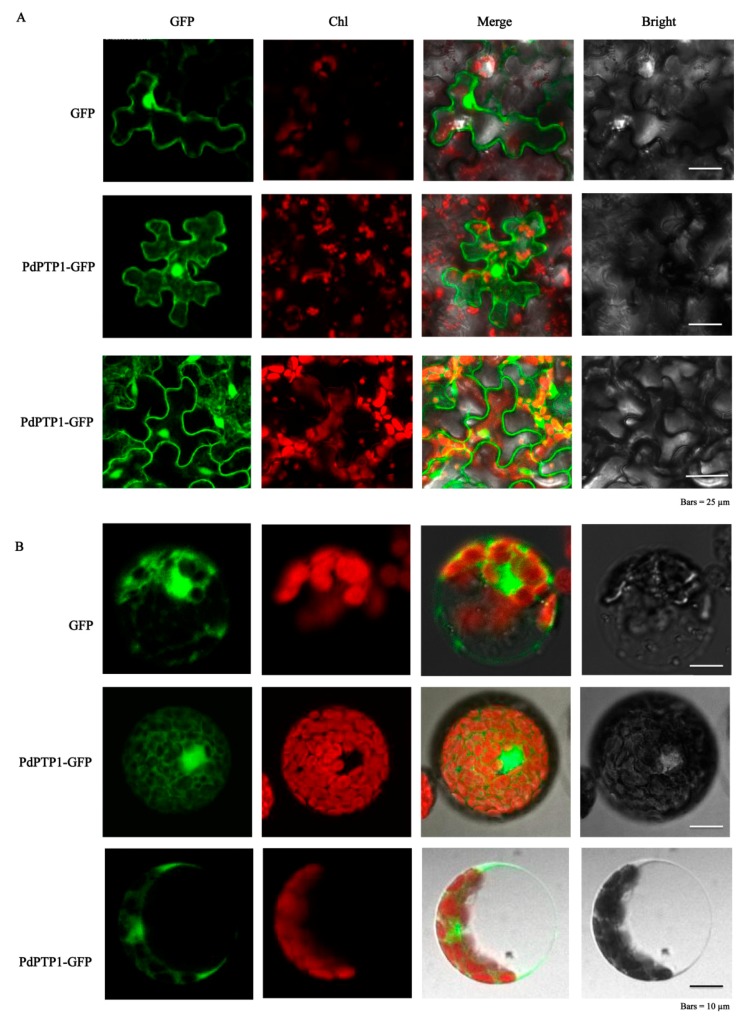
Subcellular localization of *PdPTP1.* (**A**) Subcellular localization of *pSuper:GFP* and *pSuper:PdPTP1:GFP* in transiently expressed tobacco leaves and transgenic poplar leaves (at the bottom). (**B**) Subcellular localization of *pSuper:GFP* and *pSuper:PdPTP1:GFP* in transiently expressed tobacco and poplar leaf protoplasts (at the bottom). The GFP signal was examined, using a confocal microscope. Chl indicates chloroplast. Bright field and green fluorescence images were merged overlay.

In order to investigate the subcellular localization of PdPTP1 in polar, we performed transient expression assays by using tobacco leaves, tobacco and poplar leaf protoplasts, with constructs expressing the green fluorescent protein (*GFP*)-*PdPTP1* fusion protein, and then the transformed protoplasts, tobacco, and transgenic poplar leaves were observed by laser confocal fluorescence microscopy. The green fluorescence protein was observed in both the cytosol and nucleus (Figure 2A,B), indicating that PdPTP1 is localized in cytosol and nucleus.

### 2.3. Expression Patterns of PdPTP1 in Response to Salt and Oxidative Stress

**Figure 3:**
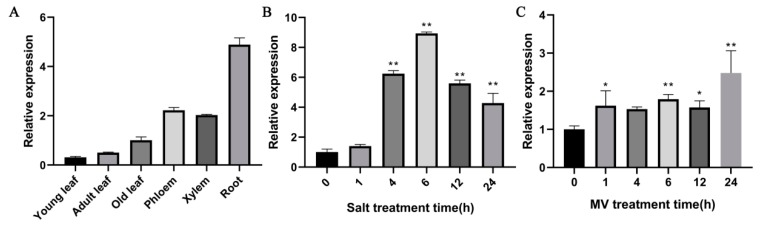
Expression profiles of *PdPTP1.* (**A**) Transcript levels of the *PdPTP1* gene in different tissues of NE19. Young leaf; adult leaf; old leaf; phloem; xylem; root. (**B**) Relative expression levels of *PdPTP1* in leaves of NE19 were measured by qRT-PCR after exposure to 350 mM of NaCl and (**C**) 100 µM of methylviologen (MV). Each column is a mean of three replicates ± SD. Asterisks indicate significant differences: ** *p <* 0.01, * *p* < 0.05.

In order to determine expression patterns of *PdPTP1*, the expression levels of *PdPTP1* in various tissues were evaluated by qRT-PCR. *PdPTP1* was expressed in all tissues tested with the highest mRNA level in roots (Figure 3A). Furthermore, we determined the transcript level of *PdPTP1* under the 350 mM NaCl treatment for 0, 1, 4, 6, 12, and 24 h. Quantitative RT-PCR analysis showed salt-stress induced *PdPTP1* expression within 1 h, reaching the highest transcript level at 6 h. The upregulation of PdPTP1 decreased afterward, up to 24 h (Figure 3B). Because salt stress could induce oxidative stress, the effect of 100 µM methylviologen (MV) on *PdPTP1* transcription also was examined. The results showed that *PdPTP1* expression was induced to increase during the entire 24 h period and reached a maximum at 24 h, after MV treatment (Figure 3C). These results suggested that the *PdPTP1* gene may be involved in salt-related stress response.

### 2.4. Identification of Transgenic Poplar

In order to investigate the function of PdPTP1 proteins, transgenic poplar overexpressing *PdPTP1* (*OxPdPTP1*) was produced by introducing the construct *pSuper*:*PdPTP1*:*GFP* into poplar 84K. Putative transgenic plants were selected for confirming the integration of transgene by PCR analysis, using the primers of the hygromycin phosphotransferase gene (Hyg) (Figure S2A). Furthermore, transgenic lines harboring *PdPTP1* were verified by qRT-PCR analysis and fluorescence signals detection (Figure S2B,C). Three transgenic lines with different level of *PdPTP1* expression (*OxPdPTP1*-1, *OxPdPTP1*-2, and *OxPdPTP1*-6) were selected for further functional analysis.

### 2.5. Overexpression of PdPTP1 Increased Sensitivity to NaCl in Transgenic Poplar

**Figure 4:**
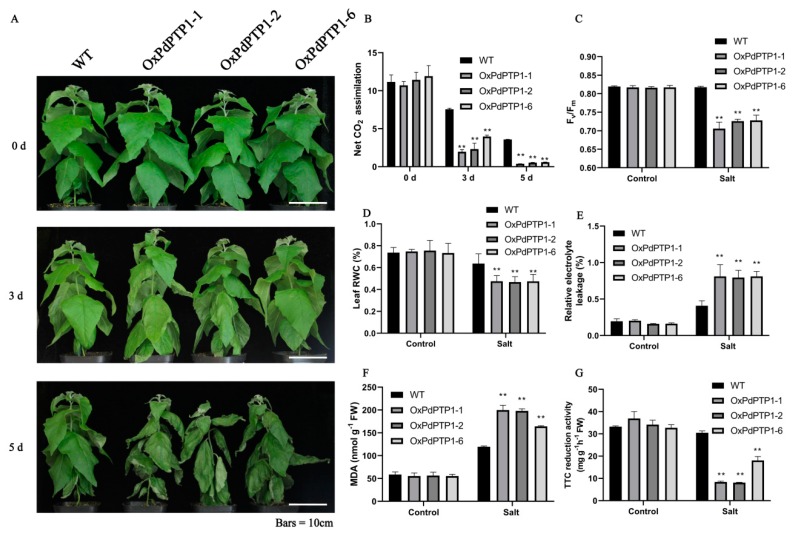
Overexpression of *PdPTP1* poplars showed decreased tolerance after exposure to 350 mM of NaCl for five days: (**A**) morphological difference, (**B**) net CO_2_ assimilation, (**C**) maximal PSII quantum yield (Fv/Fm), (**D**) leaf relative water content (RWC), (**E**) relative electrolyte leakage (REL), (**F**) malondialdehyde (MDA) in leaves, and (**G**) triphenyl tetrazolium chloride (TTC) reduction activity in roots under 0 and 350 mM of NaCl. Bar = 10 cm. Each column is a mean of three replicates ± SD. Asterisks indicate significant differences: ** *p <* 0.01.

Since *PdPTP1* was a salt induced gene, it is probable that PdPTP1 functions in a plant’s response to salt. To confirm this assumption, we next treated WT and *OxPdPTP1* poplars with 350 mM of NaCl. After five days, *OxPdPTP1* poplars showed more severe shrinking of the leaves than WT poplars (Figure 4A). Similar results were observed when *Arabidopsis* overexpressing *PdPTP1* was treated with 200 mM of NaCl (Figure S3). The Photosynthesis and Maximal PSII quantum yield (Fv/Fm) have the potential of evaluating overall quantum yield and capacity [27]. We measured photosynthetic rate and Fv/Fm of WT and *OxPdPTP1* poplars after exposure to 350 mM of NaCl. As expected, salt stress resulted in a decrease in photosynthetic rate in all lines, with the effect being most pronounced in *OxPdPTP1* poplars (Figure 4B). Changes in Fv/Fm in response to 350 mM of NaCl were also compared between the WT and *OxPdPTP1* poplars. The *OxPdPTP1* poplars leaves had relatively lower Fv/Fm than those of WT poplars after salinity exposure (Figure 4C). Furthermore, leaf RWC was measured in the *OxPdPTP1* and WT plants upon exposure to salt stress. Consistently, *OxPdPTP1* poplars exhibited an obvious decrease in leaf RWC than that in WT plants (Figure 4D). 

The REL and MDA are used as an indicator of tolerance to abiotic stresses, such as drought, salinity, and extreme temperatures [9]. To further evaluate the effects of salt stress on the performance of *OxPdPTP1* poplars, we thus compared the levels of REL and MDA in the leaves of *OxPdPTP1* poplar under 350 mM NaCl stress with those in WT. Under normal growth conditions, the physiological levels of the total REL and MDA were similar between *OxPdPTP1* and WT plants. Following salt stress, the total REL averagely increased by 52.07% in WT poplars, but 74.96%, 79.96%, and 80.00% in *OxPdPTP1*-1, *OxPdPTP1*-2, and *OxPdPTP1*-6 poplars, respectively (Figure 4E). Higher contents in the MDA were also obtained from these transgenic plants after salt treatment (Figure 4F). The effect of salt stress on plants first acts on the roots. The vitality of root directly affects plant growth and reflects the ability of plants to withstand environmental stresses. TTC reduction assay is commonly used to access the vitality of root [28]. To evaluate the effects of salt stress on the root vitality of *OxPdPTP1* poplars, TTC reduction assay was performed. Under normal condition, *OxPdPTP1* and WT roots had similar TTC reduction activity (Figure 4G). Following 350 mM NaCl stress, *OxPdPTP1* roots had lower TTC reduction activity than that of WT. Together, these results indicated that overexpression of *PdPTP1* poplars made them more sensitive to salt stress. 

### 2.6. Overexpression of PdPTP1 Poplar Increased ROS Levels and Inhibited ROS Scavenger Activities in Response to Salt Stress

**Figure 5:**
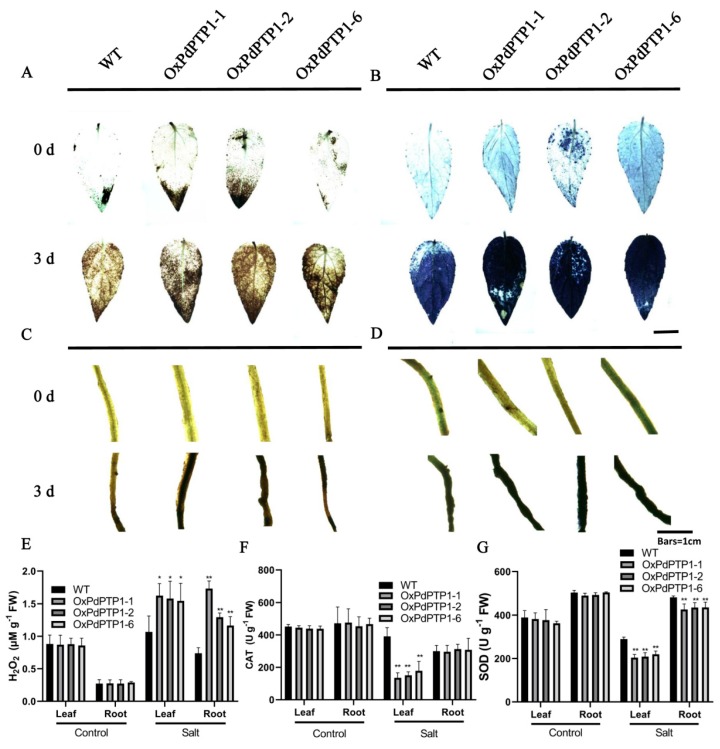
ROS contents in leaves and roots of WT and *OxPdPTP1* lines. (**A**) 3,3′ *N*-diaminobenzidine tetrahydrochloride (DAB) and (**B**) nitro blue tetrazolium (NBT) histochemical staining in leaves. (**C**) 3,3′ *N*-diaminobenzidine tetrahydrochloride (DAB) and (**D**) nitro blue tetrazolium (NBT) histochemical staining in roots. ROS accumulation was monitored by DAB and NBT for visualization of H_2_O_2_ and O_2_·^−^ produced in WT and poplar leaves and roots under the 350 mM of NaCl for zero and three days. (**E**) H_2_O_2_ content (**F**) catalase (CAT) activity (**G**) superoxide dismutase (SOD) activity in leaves and roots under 0 and 350 mM NaCl treatment for five days. Each column is a mean of three replicates ± SD. Asterisks indicate significant differences: ** *p <* 0.01 and * *p <* 0.05

For localization and quantification of H_2_O_2_ and O_2_·^−^ in vivo, WT and *OxPdPTP1* poplar leaves and roots were treated with 350 mM of NaCl for zero and three days and then stained with diaminobenzidine (DAB) (dark brown) (Figure 5A,C) and nitrotetrazolium blue chloride (NBT) (dark blue) (Figure 5B,D). The *OxPdPTP1* plants clearly exhibited higher intensities of DAB and NBT staining in leaves and roots compared with WT plants (Figure 5C,D) after 350 mM NaCl treatment for three days, indicating a higher level of H_2_O_2_ and O_2_·^−^ accumulation. Additionally, H_2_O_2_ content in the leaves and roots of *OxPdPTP1* and WT plants were measured. The result showed that H_2_O_2_ content of the leaves and roots was similar in WT and *OxPdPTP1* poplars under normal growth condition, while salt treatment increased the amount of H_2_O_2_ by an average of 17.28% and 63.21% in leaves and roots of WT poplars, respectively, but by an average of 45.09% and 79.44% in leaves and roots of *OxPdPTP1* poplars (Figure 5E). Furthermore, we examined the activities of ROS scavengers and found there was a significant decrease in CAT activity of OxPdPTP1 leaves but not roots, compared with WT (Figure 5F). SOD activity in both leaves and roots of *OxPdPTP1* poplars was lower than that of WT poplars after exposure to 350 mM of NaCl (Figure 5G). Taken together, these data indicated the high level of ROS in *OxPdPTP1* poplars under salt stress might be caused by inactivated ROS scavengers.

### 2.7. Overexpression of PdPTP1 Polar Compromised Ion Homeostasis in Response to Salt Stress

**Figure 6:**
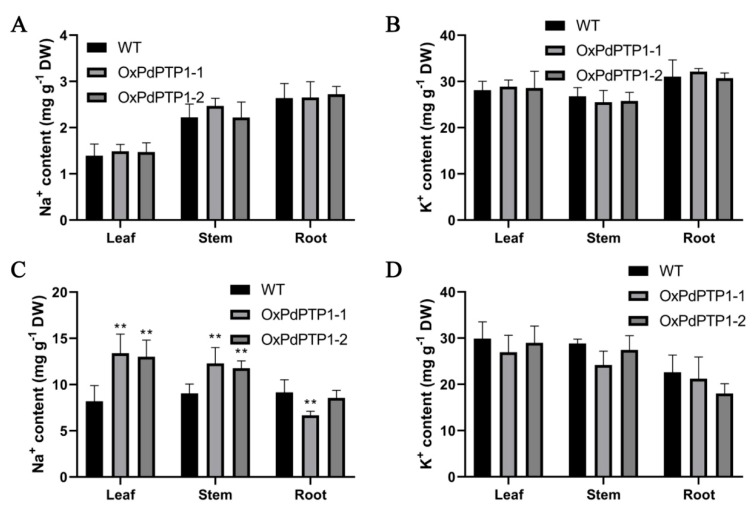
Na^+^ and K^+^ contents in WT and *OxPdPTP1* lines. (**A**) Na^+^ contents and (**B**) K^+^ contents in leaf, stem, and root under 0 mM NaCl treatment for five days. (**C**) Na^+^ contents and (**D**) K^+^ contents in leaf, stem, and root under 350 mM of NaCl for five days. Each column is a mean of three replicates ± SD. Asterisks indicate significant differences: ** *p <* 0.01.

To assess the effect of *PdPTP1* overexpression on ion homeostasis, the Na^+^ and K^+^ contents of *OxPdPTP1* and WT plants were measured after exposure to 350 mM of NaCl for five days. Under nonstress growing condition, there was no significant variation in the Na^+^ and K^+^ contents between *OxPdPTP1* and WT plants (Figure 6A,B). After NaCl treatment, Na^+^ content of *OxPdPTP1* was significantly higher than that of WT in leaves and stems but not in roots (Figure 6C). Generally, *OxPdPTP1* poplars had a slightly lower K^+^ content in stems, leaves, and roots than that in WT plants after salt treatment (Figure 6D). Taken together, our data suggested that *OxPdPTP1* negatively regulates Na^+^ and K^+^ homoeostasis under salt stress. 

### 2.8. Overexpression of PdPTP1 Reduced Salt-Stress Tolerance in Transgenic Poplar under Long-Term Salt Stress 

**Figure 7:**
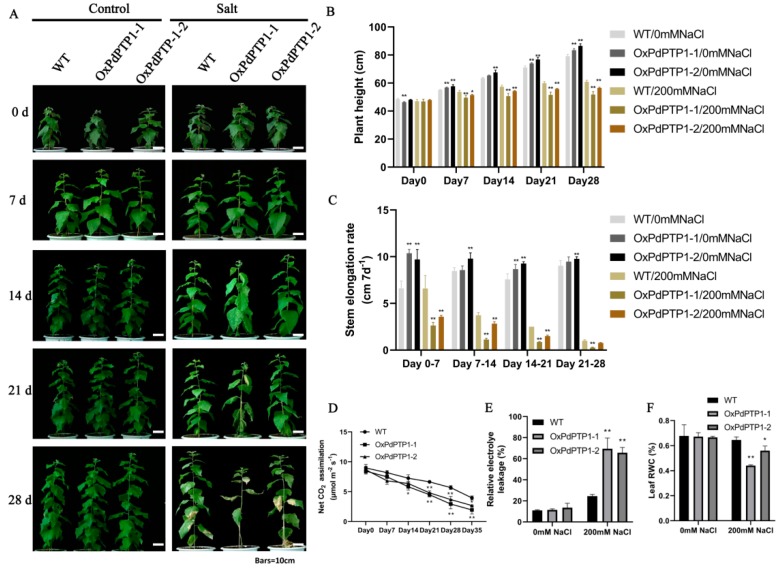
Overexpression of *PdPTP1* reduced salt tolerance in transgenic poplars under 200 mM NaCl treatment for four weeks. (**A**) Morphological differences between WT and *OxPdPTP1* lines subjected to 200 mM NaCl for four weeks. Bars = 10 cm. Measurements of (**B**) the plant height, (**C**) stem elongation rate, (**D**) net CO_2_ assimilation, (**E**) relative electrolyte leakage (REL), and (**F**) leaf relative water content (RWC) under 200 mM NaCl after four weeks. Each column is a mean of three replicates ± SD. Asterisks indicate significant differences: ** *p <* 0.01 and * *p <* 0.05.

To further explore the effects of *OxPdPTP1* on poplar during salt stress, Greenhouse experiment was applied to WT and *OxPdPTP1* plants under 0 and 200 mM NaCl condition for a four-week period. As shown in Figure 7, compared with WT plants, *OxPdPTP1* plants’ growth was severely limited under 200 mM NaCl treatment (Figure 7A). *OxPdPTP1* poplars showed a significantly lower plant height and stem height growth rate during the period of salt treatment. Interestingly, under nonstress growing conditions, *OxPdPTP1* poplars had a slightly greater plant height and stem height growth rate than WT plants (Figure 7B,C). Consistent with the growth phenotype under 200 mM NaCl treatment, *OxPdPTP1* poplars had a significantly lower photosynthetic rate than WT plants during salt stress (Figure 7D). Furthermore, the REL and leaf RWC of *OxPdPTP1* plants were similar to that of WT plants under normal growth conditions, while the *OxPdPTP1* poplars showed higher REL and lower leaf RWC than WT poplars after exposure to 200 mM of NaCl for four weeks (Figure 7E,F). 

**Figure 8:**
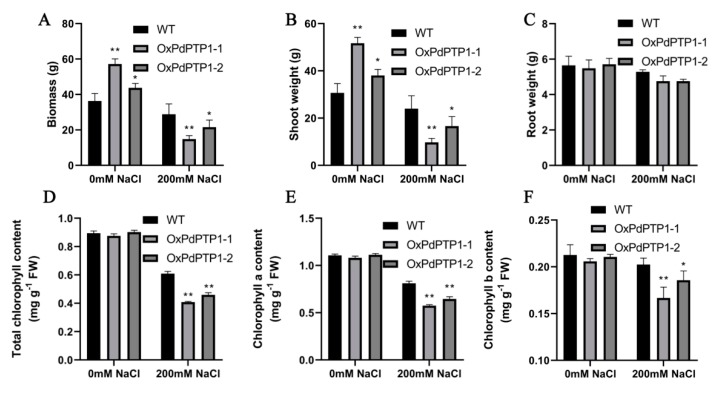
Overexpression of *PdPTP1* reduced salt tolerance in transgenic poplar of watering with 200 mM of NaCl for four weeks. (**A**) Biomass, (**B**) shoot weight, (**C**) root weight, (**D**) total contents of chlorophyll, (**E**) contents of chlorophyll a, and (**F**) contents of chlorophyll b of WT and *OxPdPTP1* after exposure to 200 mM of NaCl for four weeks. Each column is a mean of three replicates ± SD. Asterisks indicate significant differences: ** *p <* 0.01 and * *p <* 0.05.

To investigate the difference in biomass accumulation between WT and *OxPdPTP1* poplars under normal and 200 mM NaCl condition, shoot fresh weight, root fresh weight, and total fresh biomass was measured after four weeks. The results showed less total biomass accumulation and shoot weight in *OxPdPTP1* under salt conditions, compared to WT plants. However, compared with WT poplars, more total biomass accumulation and shoot weight were found in *OxPdPTP1* poplars under normal conditions (Figure 8A,B). However, the root weight showed no obvious difference between WT and transgenic poplars under either normal or salt-stress irrigation (Figure 8C). 

Under normal growth conditions, no significant difference in chlorophyll contents of WT and *OxPdPTP1* poplars was observed, but there was a significant difference when exposed to 200 mM of NaCl for four weeks (Figure 8D–F). Chlorophyll a content in *OxPdPTP1*-1 and *OxPdPTP1*-2 plants showed 52.42% and 49.08% reduction, but 31.97% in WT plants under salinity (Figure 8E). Similarly, salinity reduced chlorophyll b content of WT plants by 4.82%, whereas it reduced that of *OxPdPTP1*-1 and *OxPdPTP1*-2 plants by about 17.84% and 11.78% (Figure 8F). Consistently, the total chlorophyll content of WT plants decreased by 26.76%, but that of *OxPdPTP1*-1 and *OxPdPTP1*-2 substantially decreased by 45.81% and 42.02%, respectively, after four weeks (Figure 8D), indicating that chlorophyll of *OxPdPTP1* poplar is prone to be degraded under salinity stress.

To investigate the impact of PdPTP1 on plant photosynthetic rate, we conducted a photosynthesis-light curves experiment on *OxPdPTP1* and WT poplars under normal condition. The results showed that *OxPdPTP1* poplars had a higher photosynthetic rate than the WT poplars (Figure S4A). Both stomatal conductance (Gs) and leaf transpiration in WT and *OxPdPTP1* poplars increased with elevated light intensity, with *OxPdPTP1* poplars showing a higher Gs and transpiration (Figure S4B,C). Conversely, the instantaneous WUE value of *OxPdPTP1* poplars was lower than that of WT plants (Figure S4D). In summary, those results suggested that *OxPdPTP1* poplars showed worse growth status than WT poplars under 200 mM NaCl treatment, while they performed better under optimal condition.

### 2.9. PdPTP1 Interacts with PdMPK3, PdMAPK6 In Vivo and In Vitro

**Figure 9:**
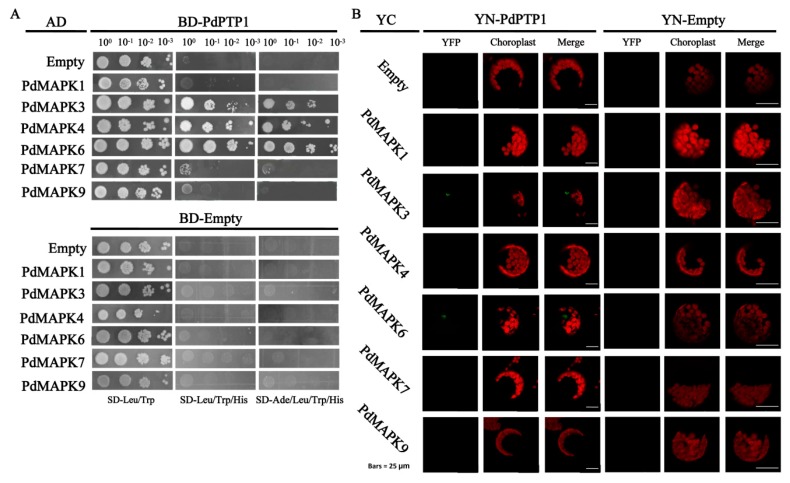
Interactions between PdPTP1 and PdMAPK3/6 in vitro and in vivo. (**A**) Proteins that interacted with PdPTP1 were identified by yeast two-hybrid assay. *PdMAPK1*, *PdMAPK3*, *PdMAPK4*, *PdMAPK6*, *PdMAPK7*, and *PdMAPK9* cDNA fragments were introduced to pGADT7 vectors and used as the prey, and *PdPTP1* cDNA fragment was introduced to pGBKT7 vectors and used as the bait, to identify the interacting proteins. Yeast strain AH109 was co-transformed with bait and prey constructs. Yeast cells with the two constructs were selected on SD-Leu/Trp (left panel). Positive clones were confirmed by growing on SD-Leu/Trp/His (middle panel) and SD-Leu/Trp/His/Ade (right panel). Combinations of *BD-PTP1* with AD-Empty, and combinations of BD-Empty with AD-Empty, *AD-PdMAPK1*, *AD-PdMAPK3*, *AD-PdMAPK4*, *AD-PdMAPK6*, *AD-PdMAPK7*, and *AD-PdMAPK9* constructs serve as negative controls. (**B**) Bimolecular fluorescence complementation (BiFC) assays of the interaction in vivo between PdPTP1 and PdMAPK3/6. Combinations of *YN-PdPTP1* (left panel) or YN-Empty (right panel) with YC-Empty, *YC-PdMPK1*, *YC-PdMPK3*, *YC-PdMAPK4*, *YC-PdMPK6*, *YC-PdMPK7*, and *YC-PdMPK9* vectors were co-transformed into tobacco leaf protoplast by means of polyethylene glycol (PEG) treatment. Interaction between *YN-PdPTP1* and YC-Empty and between YN-Empty and *YC-PdMPK1*, *YC-PdMPK3*, *YC-PdMAPK4*, *YC-PdMPK6*, *YC-PdMPK7,* and *YC-PdMPK9* (right panel) serves as negative controls. Chloroplast field and yellow fluorescence images were merged overlay. Scale bars = 25µm.

To understand the mechanism through which PdPTP1 regulates poplar salt-stress response, we performed a yeast two-hybrid assay to identify PdPTP1-interacting proteins, including PdMAPK1, PdMAPK3, PdMAPK4*,* PdMAPK6, PdMAPK7, and PdMAPK9. Among the six candidates we identified, PdMAPK3/4/6 interacted strongly with PdPTP1 (Figure 9A). We used a BiFC assay to further address the spatial specificity of PdPTP1–PdMAPKs complex formation. However, we cannot detect a sign of interaction between PdPTP1 and PdMAPK4. Additionally, the BiFC assay also showed that no sign of interaction could be detected between PdPTP1 and PdMAPK1, PdMAPK7, and PdMAPK9, or any of the negative controls (Figure 9B).

## 3. Discussion

Poplar is widely distributed throughout the world and serves as a model organism for tree molecular biology and forest biotechnology because of its relatively small genome, rapid growth rate, ease of vegetative propagation, and genetic transformation [29,30]. Apart from scientific importance, poplar is used for the construction industry, paper making, and biofuel production and thus has significant economic value [31]. As a perennial tree species, poplar trees are routinely exposed to complex environmental stresses, such as salt, drought, and low temperature during their long life span, and these stresses adversely affect their growth and survival [32,33]. To meet the increasing demand of poplar productivity, it is important for us to understand molecular responses to stress in poplar and further improve stress tolerance through genetic engineering. In this work, we show that PdPTP1 is a negative component in the salt signaling pathway. *PdPTP1* overexpression poplars displayed hypersensitivity to salt stress. 

### 3.1. PdPTP1 is a Tyr-Specific PTP in Poplar

The conserved catalytic signature (I/V)HCXAGXXR(S/T)G defined the large PTP superfamily, which now, in addition to DsPTPs, includes Tyr-specific PTPs that only dephosphorylate tyrosine [34]. In the present study, PdPTP1 contains the (I/V)HCXAGXXR(S/T)G motif in the catalytic domain characteristic of PTPs superfamily (Figure S1). Moreover, the PdPTP1 harbors two conserved motifs which are present in all members of Tyr-specific PTPs but was not detectable in the sequence of any other DsPTPs family: KNRY motif necessary to permit the access of phosphotyrosine residues to the active site and WPD motif essential for the phosphotyrosine activity in all eukaryotes (Figure S1) [35]. Another unique sequence signature for Tyr-specific PTPs in plants, IHNT motif, also appears in the amino acid sequence of PdPTP1 (Figure 1B) [36]. Taken together, the presence of these motifs clearly places the PdPTP1 within the Tyr-specific PTPs family. 

### 3.2. PdPTP1 Overexpression Reduced Tolerance to Salt Stress in Poplar due to the Impaired Ion and ROS Homeostasis

In plants, PTP1 is involved in various stresses, such as salt, oxidative, osmotic, pathogen response, wounding, and ABA signaling [14,19,24,36,37]. Our results found that the transcript of *PdPTP1* was induced by NaCl and oxidative treatment (Figure 3B,C), and this finding is consistent with previous studies that the transcript of *AtPTP1* was upregulated by high salinity and H_2_O_2_ [14,38]. These results indicate that PdPTP1 protein may play an important role in response to salt stress. For further understanding of *PdPTP1* function under salt stress, we treated *OxPdPTP1* poplar with salt stress and found that *OxPdPTP1* poplar displayed hypersensitivity to salt (Figure 4); similar results were obtained in cotton (Gossypium spp.), in which GhDsPTP3a, a member of PTPs, reversely regulated salt tolerance in cotton [39], indicating a conserved function of PTPs in regulating salt response.

### 3.3. PdPTP1 Overexpression Reduced Salt Tolerance in Poplar due to the Impaired ROS Homeostasis

Salt stress leads to the accumulation of high levels of ROS. ROS is highly reactive and can lead to impaired physiological function through cellular damage of phospholipids [7,40]. The data obtained in the present study showed that the *OxPdPTP1* plant suffered remarkable ROS toxicity under salt stress (Figure 5), resulting in higher REL and more accumulation of MDA than WT plant (Figure 4E,F), which cause more serious membrane damage and thus accelerated the *OxPdPTP1* plant’s aging and death. In order to avoid destruction of ROS, plants have developed enzymatic components such as SOD and CAT [2]. In the present study, the CAT and SOD activities in *OxPdPTP1* plants were lower than in the WT plant (Figure 5D,E), which suggested that obvious lower activity of antioxidant enzymes rendered the *OxPdPTP1* poplar to suffer more serious ROS toxicity and thus reduced salt tolerance in *OxPdPTP1* poplar.

### 3.4. PdPTP1 Overexpression Reduced Salt Tolerance in Poplar due to the Impaired Ion Homeostasis

Maintaining cellular ion homeostasis is an important adaptive trait of salt-tolerant plants during salt stress. A suitable K^+^/Na^+^ in the cytoplasm prevents cellular damage and nutrient deficiency [2]. In this present study, on one hand, more Na^+^ accumulates in leaves and stems, but less in roots of *OxPdPTP1* poplars than WT poplars under salinity condition (Figure 6C). On the other hand, lower K^+^ was observed in leave, stems, and roots of *OxPdPTP1* poplars (Figure 6D), which suggested that salt sensitive phenotype of *OxPdPTP1* plants result from impaired ion homeostasis. This result is consistent with the previous report that GhDsPTP3a reduces Na^+^ efflux and thus causes plant hypersensitivity to salt stress [39].

Salt stress poses a serious threat to photosynthesis and the electron transport system [41]. Excessive cytoplasmic Na^+^ gives rise to imbalanced cellular ion contents, causing reduced maximum photochemistry efficiency of PSII and substantial reduction in photosynthesis [42,43]. In this study, we observed that overexpression of *PdPTP1* poplars significantly decreased the maximal photochemical yield of PSII (Fv/Fm) in *OxPdPTP1* poplars compared with WT poplars after salt treatment (Figure 4C). The ratio Fv/Fm was used as parameters to evaluate the maximum photochemical efficiency of PSII in the dark-adapted state and decline of Fv/Fm value reflects the occurrence of photoinhibition of photosynthesis [44]. Lower Fv/Fm indicated that light-use efficiency of the *OxPdPTP1* plant decreased under salt-stress conditions and explains why the *OxPdPTP1* plant has a lower photosynthetic rate than the WT plant (Figure 4B). Taken together, these results suggested that the photosynthetic machinery is damaged by deleterious salt effects, probably due to the excessive Na^+^ accumulation in *OxPdPTP1* poplars.

### 3.5. The Hypersensitivity of OxPdPTP1 Poplar to Salt Is Associated with PdMPK3/6

In plants, MAPK cascades are universal signal transduction modules, which can sense stress signals and transduce them into suitable responses [45]. Many data suggested that MAPKs is rapidly activated in plants exposed to a variety of abiotic and biotic stresses, including salt, cold, drought, UV-irradiation, wounding, and pathogen [46,47]. Among them, MPK3/6 has been well studied in salt stress [48,49,50]. For example, MPK3/6 phosphorylate and activate HSFA4A, thereby controlling ROS homeostasis and positively regulating salt-stress responses [13]. AtMPK6 activated by PLD1-derived phosphatidic acid (PA) phosphorylates and activates the SOS1 Na^+^/H^+^ antiporter activity and thus enhances salt tolerance [11]. These reports indicated that MPK3/6 improve salt tolerance by re-establishing cellular ROS and ion homeostasis. MAPKs can be activated by phosphorylating Thr and Tyr residues in an activation loop of MAPK by dual-specificity MAPKKs. On the contrary, dephosphorylation of Thr or/and Tyr bring the enzymes back to the inactive state. This can be achieved by PTPs and DsPTPs [24]. In *Arabidopsis*, AtPTP1 had been shown to be able to dephosphorylate and deactivate AtMPK3/6 either in vitro or in vivo [19,26]. The tight regulation of MAPKs by MAPKKs and PTPs is a prerequisite to the fine-tune physiological outcome of signaling produce specific and adequate physiological responses [26]. In the present study, we found that PdPTP1 interacted with PdMPK3/6 in vitro and in vivo (Figure 7A,B), and transgenic poplar of overexpressing *PdPTP1* showed sensitivity to salt due to the toxicity of ion and ROS. Therefore, we suppose that the overexpression of PdPTP1 may be associated with decreased levels of active MAPK3/6 in response to salinity, which result in disrupted ion and ROS balance of poplars under salt stress and therefore exhibited a salt-hypersensitive phenotype. In particular, *PdPTP1* mRNA was induced by salt, indicating PdPTP1 may act in a feedback loop to avoiding hyperactivation of PdMAPK3/6 and thus guarantee a fine-tune physiological response. Similarly, in fission yeast, Pyp2, which encodes a tyrosine-specific phosphatase, functions in a feedback loop to inactivate Styl MAPK kinase, following exposure to osmotic stress [51]. However, the precise role of PdPTP1 in MAPK3/6 dephosphorylation is under further investigation. 

**Figure 10:**
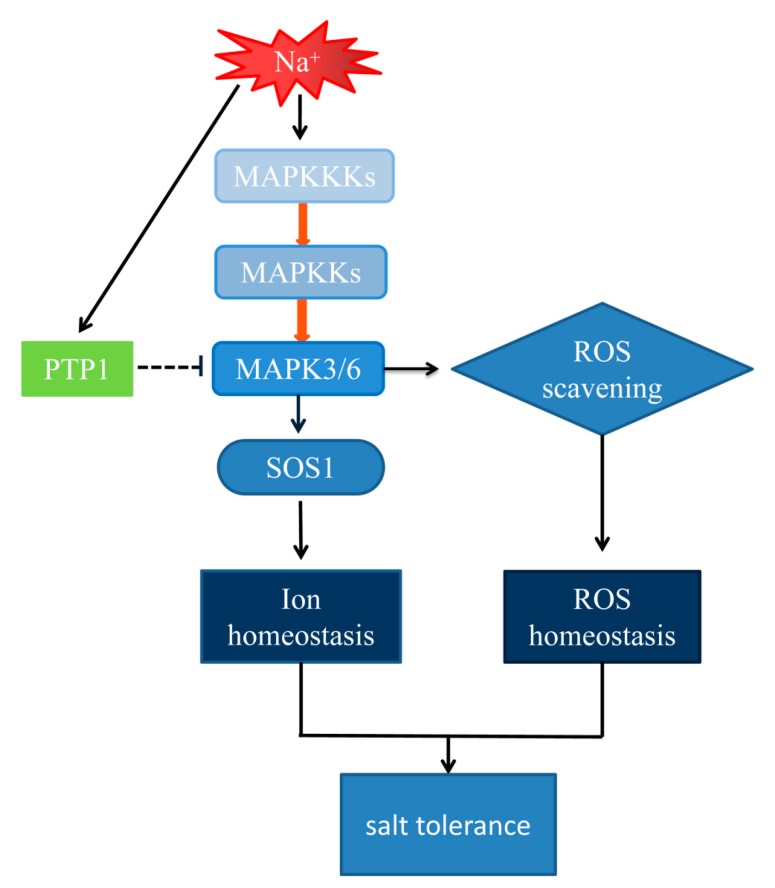
A model for the putative pathway of regulation of salt stresses by PTP1. Under salt stress, the MAPKKK-MAPKK-PdMAPK3/6 cascades are activated. Activated PdMAPK3/6 may be directly dephosphorylated by PdPTP1. PdPTP1 may cooperate with PdMAPK3/6 to regulate ion homeostasis and, on the other hand, scavenge ROS to avoid oxidative damage in response to salt. Direct stimulatory modification indicated with the red arrows, Stimulatory modification indicated with the black arrows, Tentative inhibitory modification indicated with the dot T-bar.

Taken together, we propose a model for the putative pathway of regulation of salt stress by PdPTP1 (Figure 10). Under salt stress, the MAPKKK-MAPKK-PdMAPK3/6 cascades are activated. Activated PdMAPK3/6 may be directly dephosphorylated by PdPTP1. PdPTP1 may cooperate with PdMAPK3/6 to regulate ion homeostasis and, on the other hand, scavenge ROS, to avoid oxidative damage in response to salt.

## 4. Material and Methods

### 4.1. Plant Materials and Stress Treatments 

*Populus alba × P. glandulosa* (84K) seedlings were cultured in half-concentrated Murashige and Skoog (MS) medium supplemented with 0.05 mg/L of IBA and 0.05 mg/L of NAA (Sigma, St. Louis, MO, USA) [52], and NE19 plantlets were cultured in MS medium in a controlled environment growth room (temperature: 25 °C:20 °C, dark:light; relative humidity: 45%; photoperiod: 16 h:8 h, light:dark). Various abiotic stress was conducted on 60-day-old NE19 seedlings, to verify the expression patterns of *PdPTP1* in different tissues, salinity stress, and oxidative stress. For oxidative stress, 100 µM of Methylviologen (MV) (Sigma, 856177) was sprayed once on the leaves of NE19. The leaves of treated poplars were harvested at 0, 1, 4, 6, 12, and 24 h, after MV treatment. For salinity stress, NE19 plants were subjected to 350 mM of NaCl. The leaves of treated poplars were harvested at 0, 1, 4, 6, 12, and 24 h, after salinity treatment. The harvested samples were immediately frozen in liquid nitrogen and stored at −80 degrees.

### 4.2. RNA Extraction and Quantitative Real-Time Polymerase Chain Reaction 

The leaves of control and treated poplars, as described above, were collected to extract total RNA by using the CTAB reagent, a method described previously [53]. Then, cDNA was created by 1 µg of RNA, using the Prime Script™ RT reagent Kit with gDNA Eraser (Perfect Real Time, Takara, RR047Q, Dalian, China), according to manufacturer’s instructions. The quantitative real-time polymerase chain reaction (qRT-PCR) was performed, as previously with methods [54], using the TB Green^®^ Premix Ex Taq™ GC (Perfect Real Time, Takara, RR071Q, Dalian, China) with gene-specific primers and the internal control gene (*ACTIN*). The relative value for the expression level of each gene was calculated by the 2^−ΔΔCt^ method [55]. Primers were designed by using Primer 5.0 (Sigma-Aldrich Corp, St. Louis, MO, USA) and the list in Table S1. 

### 4.3. Salt-Stress Tolerance Evaluation of Transgenic Poplars

All of the plants (height 20–30 cm) were grown in suitably sized pots (10 cm long, 10 cm wide, and 10 cm high) of the same soil (nutrient soil, peat, and vermiculite 1:1:1), and each pot had a tray. In order to investigate the functional role of PdPTP1 in response to salt stress, potted WT lines and *OxPdPTP1* lines were treated with short-term salt treatment and long-term salt treatment. To short-term salt treatment, WT lines and *OxPdPTP1* lines with similar size and growth status were chosen and subjected to 0 or 350 mM of NaCl for 5 days. Net photosynthetic rate, transpiration, stomatal conductance and intercellular CO_2_ concentration in the 9th to 11th leaves of *OxPdPTP1* and WT poplars were measured by the Li-6400 Photosynthesis System (Li-Cor, Lincoln, NE, USA) at day 0, day 3, and day 5. Five days after exposure to 350 mM of NaCl, photosynthetic activity in the 10th to 13th leaves of WT lines and *OxPdPTP1* lines was monitored by Maximal PSII quantum yield (Fv/Fm) values, using a PAM chlorophyll fluorometer (PAM100) after 20 min of dark adaptation. Relative electrolyte leakage (REL), leaf relative water content (RWC), Malondialdehyde (MDA) content, and root reduction activity of WT lines and *OxPdPTP1* lines were determined as described through previous protocol [54]. Root reduction activity was analyzed by the triphenyl tetrazolium chloride (TTC) method [56]. For the long-term salt treatment, WT lines and *OxPdPTP1* lines with similar size and growth status were chosen and subjected to 1/8 MS solution supplemented with or without 200 mM of NaCl, every 4 days for 4 weeks. The height of WT lines and *OxPdPTP1* lines was measured weekly. Net photosynthetic rate, transpiration, stomatal conductance and intercellular CO_2_ concentration in the 9th to 11th leaves of WT plants and transgenic lines were measured by the Li-6400 Photosynthesis System (Li-Cor, Lincoln, NE, USA) weekly. After 4 weeks, leaf RWC, REL, shoot biomass, root biomass, and chlorophyll content of each poplar were measured. 

### 4.4. Measurement of Na^+^ and K^+^ Contents

At the end of 350 mM of NaCl treatment for 5 days, the leaves, stems, and roots were harvested for Na^+^ and K^+^ assay, as described previously [57,58]. Briefly, the samples were dried at 80 °C, for 2 day, milled to fine powder, weighed, and digested with concentrated sulfuric acid. The Na^+^ and K^+^ contents were determined by using an atomic absorption spectrometer (TAS-986; Purkinje General Instrument Co., Beijing, China).

### 4.5. Subcellular Localization Analysis

For subcellular localization of PdPTP1, *pSuper*:*PdPTP1*:*GFP*, and *pSuper*:*GFP* fusion constructs were transformed into tobacco epidermal cells by the previously published protocol [59] and tobacco and poplar protoplasts by means of polyethylene glycol (PEG) treatment [60]. The transformed protoplasts, tobacco, and transgenic poplar leaves were observed by laser confocal fluorescence microscopy (Leica TCS SP8; Leica, Wetzlar, Germany). The LAS-AF software was used to record the images. 

### 4.6. Yeast Two-Hybrid

Yeast two-hybrid analysis was performed, using the protocol described by the previously published protocol [61]. The full lengths of *PdMAPK1*, *PdMAPK3*, *PdMAPK4*, *PdMAPK6*, *PdMAPK7*, and *PdMAPK9* cDNA fragments were introduced to pGADT7 vectors (Clontech, Mountain View, CA, USA) and used as the prey, and the full length of *PdPTP1* coding sequences cDNA fragment was introduced to pGBKT7 vectors (Clontech) and used as the bait, to identify the interacting proteins. Yeast strain AH109 was co-transformed with bait and prey constructs. Yeast cells with the two constructs were selected on minimal medium without leucine (Leu) and tryptophan (Trp) medium. Positive clones were confirmed by growing on minimal medium containing 6 mM of 3-amino-1,2,4-triazole without Leu, Trp, and histidine (His), and minimal medium without Leu, Trp, His, and adenine (Ade). 

### 4.7. Bimolecular Fluorescence Complementation (BiFC) Assays

For BiFC assay, *PdPTP1* coding sequences were recombined into the pSPYNE vector to produce *YN-PdPTP1*, *PdMAPK1*, *PdMAPK3*, *PdMAPK4*, *PdMAPK6*, *PdMAPK7*, and *PdMAPK9* coding sequences, which were then recombined into pSPYCE vectors, to produce *YC-PdMPK1*, *YC-PdMPK3*, *YC-PdMAPK4*, *YC-PdMPK6*, *YC-PdMPK7*, and *YC-PdMPK9* [62]. Co-transformation of *YN-PTP1* or *YN*-Empty, in combination with *YC*-Empty, *YC-PdMPK1*, *YC-PdMPK3*, *YC-PdMAPK4*, *YC-PdMPK6*, *YC-PdMPK7*, and *YC-PdMPK9*, was also performed in tobacco protoplasts by means of PEG treatment. The fluorescence was detected by using confocal microscopy (Leica TCS SP8; Leica, Wetzlar, Germany). The LAS-AF software was used to record the images. 

### 4.8. Phylogenetic and Domain Analysis of PdPTP1

The MtPTP1, NtPTP1, and LePTP1 protein sequences were obtained from NCBI (National Coalition Building Institute, https://www.ncbi.nlm.nih.gov/), and sequences data of the other proteins shown in the diagram can be found in the GenBank database, under the accession numbers (Table S2) The phylogenetic tree of PTPs was established with MEGA X by the Maximum Likelihood method, and the maize calcium-dependent protein kinase (ZmCDPK2; AAA69507) was used as an outgroup [63]. The alignments were generated by the multiple sequence alignment by the Florence Corpet website (http://multalin.toulouse.inra.fr/multalin/multalin.html) [64].

### 4.9. Statistical Analyses 

The experiment was repeated at least three times, independently. The results are presented as the means ± SDs. Data were analyzed through LSD multiple range tests in the one or two way ANOVA program of SPSS (IBM SPSS17.0). Significance was defined as *p* < 0.05 (*) and *p* < 0.01 (**).

## Supplementary Materials

Supplementary materials can be found at https://www.mdpi.com/1422-0067/21/3/1065/s1.
