# Supplementary material for: Poplar PdPTP1 Gene Negatively Regulates Salt Tolerance by Affecting Ion and ROS Homeostasis in Populus
Source: Int J Mol Sci. 2020 Feb 5;21(3):1065. doi: 10.3390/ijms21031065 (PMC7037657; doi:10.3390/ijms21031065)
Supplement: Supplementary file 1 [file ijms-21-01065-s001.pdf]

Table S1 qRT-PCR and PCR primers used in this study

Gene name/ ID	Primers (F: Forward, R: Reverse)
qRT-qPCR	
PdNhaD1/JX981308	F: GGACTCTTCTTTGGGTGGTTGGTTT<br>R: GCTTGCGGTATTCTGATGGAGGTAC
PdSOS1/Potri.010G100900	F: CAAGCATTTTCCCTCAGAGGATG<br>R: CACTGCCACCAATAAAGTCGT
UbQ/*Potri.014G115100*	F: AGACCTACACCAAGCCCAAGAAGAT<br>R: CCAGCACCGCACTCAGCATTAG
PdPTP1/Potri.013G115400	F: GAATTCTCTCCCGATCCGCC<br>R: TTGTGCGATTCTCTTCGGGG
PCR	
PdPTP1/Potri.013G115400	F: ATGGCCTCCTCCTCCGCT<br>R: ACTGTTCATCTCCGAGATGAGATCT
PdMPK1/Potri.002G032100	F: CATAAGACCAATAAAGGGAATGGC<br>R: GTGCTCGAATTGAAGGCGATG
PdMPK3/Potri.001G271700	F: CGAAACAGACGATCGGAGATG<br>R: CGGGATTGGTAGTGCTAAAGG
PdMPK4/Potri.002G162500	F: ACTGGACGGACTGCTTAATCA<br>R: TTGTTGTCCATTGTAACCATGGG
PdMPK6/Potri.017G010200	F: GGCATGGAAGGTGGAAGTC<br>R: ACAATGAGTCGACCAGCGTG
PdMPK7/Potri.005G119500	F: ACATTACAAGCAACCAGGTGA<br>R: AAGCATGGCGTTGAGAGAA
PdMPK9/Potri.012G048600	F:AGTGACAAGATAGGGGGAGAGAG<br>R: GCACCCACTATCCACATAAAGC

Table S2 Accession numbers of PTPs from eukaryotes

Gene name	ID
Arabidopsis thaliana AtPTP1	NP_177331
Glycine max GmPTP1	CAA06975
Pisum sativum PsPTP1	CAA06615
Ricinus communis RcPTP1	XP_002534020
Medicago truncatula MtPTP1	XP_013469551
Nicotiana tobacum NtPTP1	XP_016470867
Lycopersicon esculentum LePTP1	XP_004241817
Phalaenopsis amabilis PaPTP1	GU119901
Oryza sativa OsPTP1	ABA96592
Oryza sativa OsPTP2	ABA91789
Zea mays ZmPTP1	NP_001149088
Homo sapiens HsPTP1B	P18031
Rattus norvegicus RnPTP18	NP_001013129
Gallus gallus GgPTP11	NP_990299
Xenopus laevis XlPTP11	Q92124
Drosophila melanogaster DmPTP	P29349
Dictyostelium discoideum DdPTP1	XP_644525
Saccharomyces cerevisiae ScPTP1	P25044

Figure S1

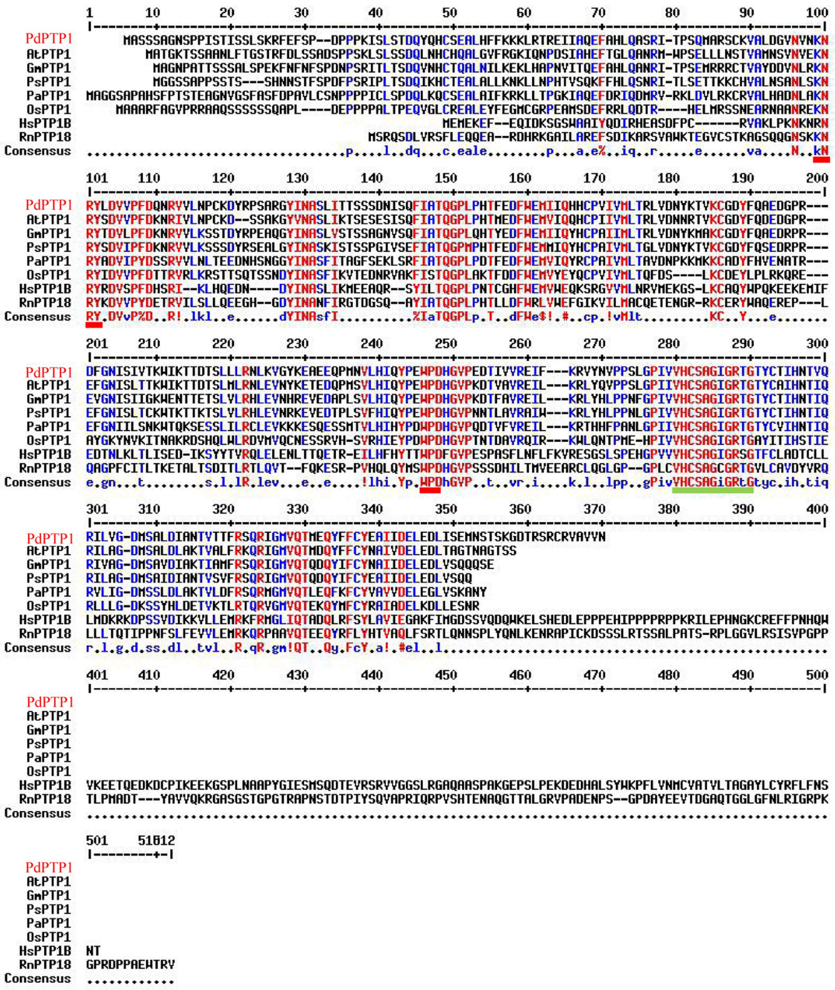

Figure S1. Comparison of the PdPTP1 protein sequence with a number of PTPs from eukaryotes. Multiple alignment of the amino acid sequences of Tyr specific PTPs from plants, human, and rat. The conserved cysteine and arginine residues within the predicted PTP active site (I/V)HCXAGXXR(S/T)G are marked with green underline. The KNRY and the WPD motifs specific to Tyr specific PTPs are highlighted in red underline.

Figure S2

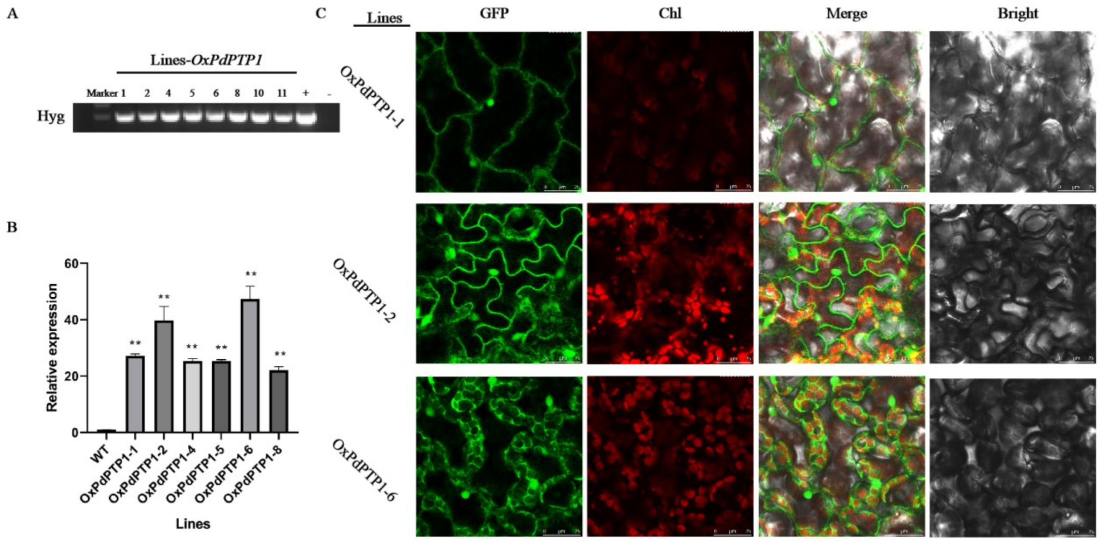

Figure S2. Confirmation of *PdPTP1* over-expressing plants. (A) PCR confirmation of independently regenerated Hygromycin-resistant lines. (B) The transcript levels of *PdPTP1* in different over-expression lines analysed by qRT-PCR. (C) The green fluorescence signals of GFP-*PdPTP1* fusion protein in different over-expression lines. Scale Bars = 25μm. Each column represents an average of three replicates, and bars indicate SDs. ** indicate significant differences in comparison with the control at *P*<0.01.

Figure S3

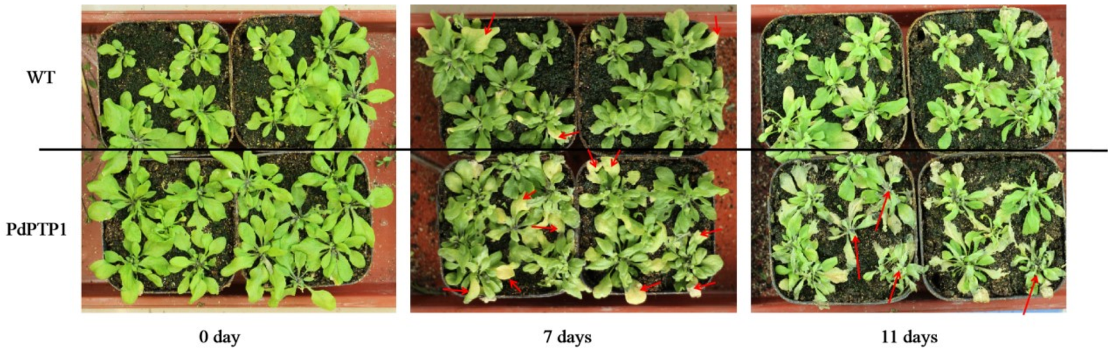

Figure S3. Salt tolerance assays in transgenic Arabidopsis lines over-expressed *PdPTP1*. Arabidopsis seedlings were irrigated with 200 mM NaCl at 4-day interval and the photos were taken at 0, 7 and 11 days after salt treatment.

Figure S4

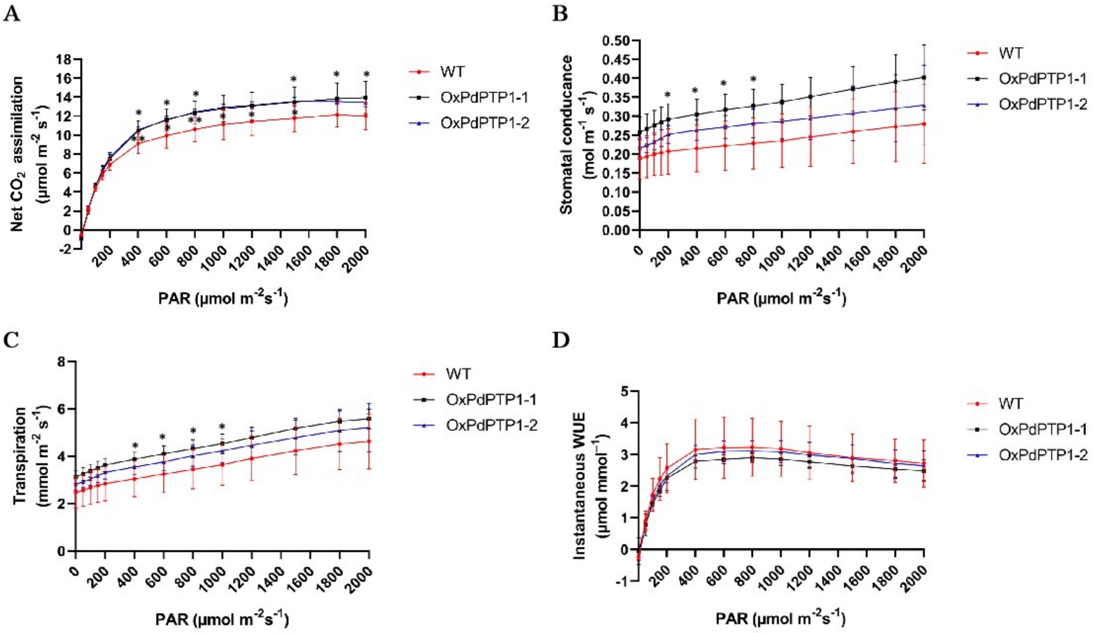

Figure S4 Light response curves were measured in WT, *OxPdPTP1*-1 and *OxPdPTP1*-2 poplar in the same greenhouse conditions. (A) A-light curve. (B) Gs-light curve. (C) Transpiration-light curve. (D) Instantaneous WUE-light curve. Each column represents an average of three replicates, and bars indicate SDs. ** and * indicate significant differences in comparison with the control at $P<0.01$ and $P<0.05$, respectively.
